# Case report of severe unilateral posterior scissors bite in children treated with clear aligners

**DOI:** 10.3389/fdmed.2026.1835728

**Published:** 2026-06-22

**Authors:** Junji Xu, Hongyu Chen, Yiduo Shao, Lanqiu Lv

**Affiliations:** The Department of Dentistry of Ningbo University Affiliated Women and Children's Hospital, Ningbo, China

**Keywords:** child malocclusion, clear aligners, early interceptive orthodontics, mandibular rotation, posterior scissors bite

## Abstract

Posterior scissors bite is a clinically common severe malocclusion, which often leads to impaired masticatory function, unilateral mastication habit and progressive facial asymmetry. This case report presented an 8-year-old boy with severe unilateral posterior scissors bite in mixed dentition, who received early interceptive orthodontic treatment with bracket-free clear aligners combined with alternating intermaxillary traction. The treatment procedures included staged expansion of dental arch width, uprighting of severely lingually inclined molars, and complete correction of posterior scissors bite. Due to poor patient compliance, treatment was interrupted and occlusal relapse occurred; thus, the traction protocol was revised and resumed. During the whole course, standardized tooth tapping training was applied to control posterior vertical height and prevent mandibular clockwise rotation. After systematic treatment, the posterior scissors bite was completely corrected, accompanied by significant improvements in dental arch morphology, dental midline alignment, anterior overjet and overbite.

**Conclusion:**

For strictly indicated cases, clear aligners combined with alternating intermaxillary traction can effectively correct severe unilateral posterior scissors bite in children, with the advantages of low discomfort and superior esthetic performance, which is worthy of clinical promotion. Nevertheless, high patient compliance must be emphasized in complex pediatric cases to avoid occlusal relapse caused by treatment interruption.

## 1 Introduction

Posterior scissors bite is a prevalent malocclusion, categorized into buccal and lingual crossbite types. Severe posterior scissors bite is defined as the lingual oblique surface of maxillary distal teeth occluding with the buccal oblique surface of mandibular distal teeth without effective occlusal contact. This condition not only compromises masticatory function and periodontal health, but also adversely affects temporomandibular joint (TMJ) growth and development; in severe cases, it results in irreversible facial asymmetry (1).

Compared with anterior crossbite, posterior scissors bite is insidious in the early stage and easily neglected by parents. Long-term abnormal occlusal interference induces functional mandibular displacement and abnormal condylar remodeling, making early interceptive intervention extremely critical. Timely correction of posterior scissors bite can restore masticatory efficiency, eliminate unilateral chewing habit, facilitate balanced bilateral TMJ development, and block the progression of facial asymmetry (2).

At present, standardized orthodontic protocols for severe unilateral complete posterior scissors bite in children are rarely reported, especially for complex cases complicated with extensive primary molar caries and severe lingual inclination of permanent molars. Relevant clinical experience remains limited. This article reported one severe unilateral posterior scissors bite case treated in The Affiliated Women and Children's Hospital of Ningbo University, with a detailed analysis of cephalometric changes, treatment procedures, biomechanical mechanism, therapeutic effects and limitations, aiming to provide reference for clinical similar cases.

## 2 Case report

### 2.1 General patient information

**Figure 1:**
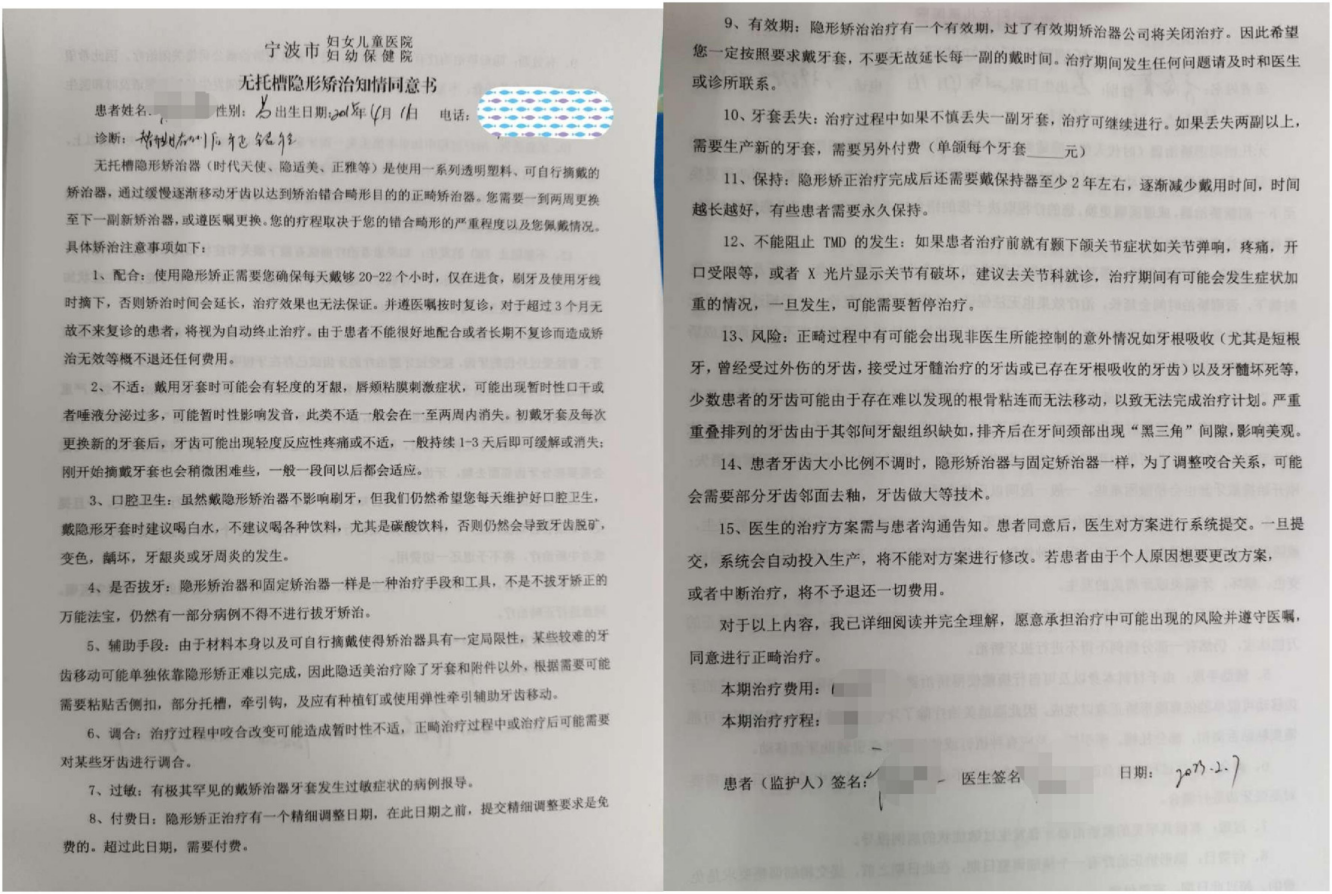
Patient informed consent form.

An 8-year-old Han male child, height 132 cm, weight 27 kg, presented for orthodontic consultation in February 2023. The guardians signed informed consent for the publication of case data and clinical images for academic use (Figure 1).

Chief complaint: Irregular alignment of right posterior teeth, inability to perform normal mastication, requesting orthodontic correction.

Personal history: No oral bad habits such as thumb sucking, lip biting or long-term unilateral chewing.

Past medical history: No systemic diseases, no trauma or surgical history.

Family history: No first-degree relatives with severe malocclusion or hereditary craniofacial deformities.

Psychological history: Normal mental status, no social inferiority or resistance to orthodontic treatment. No previous orthodontic treatment history.

### 2.2 Clinical examination

#### 2.2.1 Extraoral examination

Mild bilateral facial asymmetry, with the right facial contour slightly smaller than the left. The lower facial height was within normal range. Lateral profile presented straight facial type with slightly deep labiomental fold.

#### 2.2.2 Intraoral examination

Mixed dentition stage: Maxillary central incisors (11, 21) and mandibular anterior teeth (32–42) had erupted; primary maxillary lateral incisor (52) was congenitally missing; permanent maxillary right lateral incisor (12) was unerupted. Anterior teeth presented Class II deep overjet and Class I deep overbite. Severe unilateral posterior scissors bite was found on the right side involving primary molars (53, 54, 55) and permanent first molar (16). Primary mandibular right second molar (85) and permanent mandibular right first molar (46) showed severe lingual inclination. Left posterior molars presented neutral occlusion. The mandibular dental arch was constricted. A 1 mm diastema existed between 11 and 21, and mild mandibular anterior crowding (1 mm). The mandibular dental midline deviated 2 mm to the right. Primary molars (55, 65, 75, 85) received filling treatment previously, with secondary caries observed at the margin of 65, 75 and 85 restorations.

#### 2.2.3 TMJ and periodontal examination

Mouth opening distance was 38 mm with straight and symmetrical opening path. No joint tenderness or clicking was detected bilaterally. Periodontal condition was good: no gingival redness, swelling, periodontal pocket or alveolar bone resorption, with acceptable oral hygiene.

#### 2.2.4 Imaging and cephalometric examination

Panoramic radiograph: 12 primary teeth remained, no periapical lesions in the whole dentition; permanent tooth germs developed normally, no impacted teeth.

CBCT: No organic lesions of bilateral TMJ; condylar morphology was symmetrical, bone structure continuous, joint space uniform without absorption or hyperplasia.

**Figure 2:**
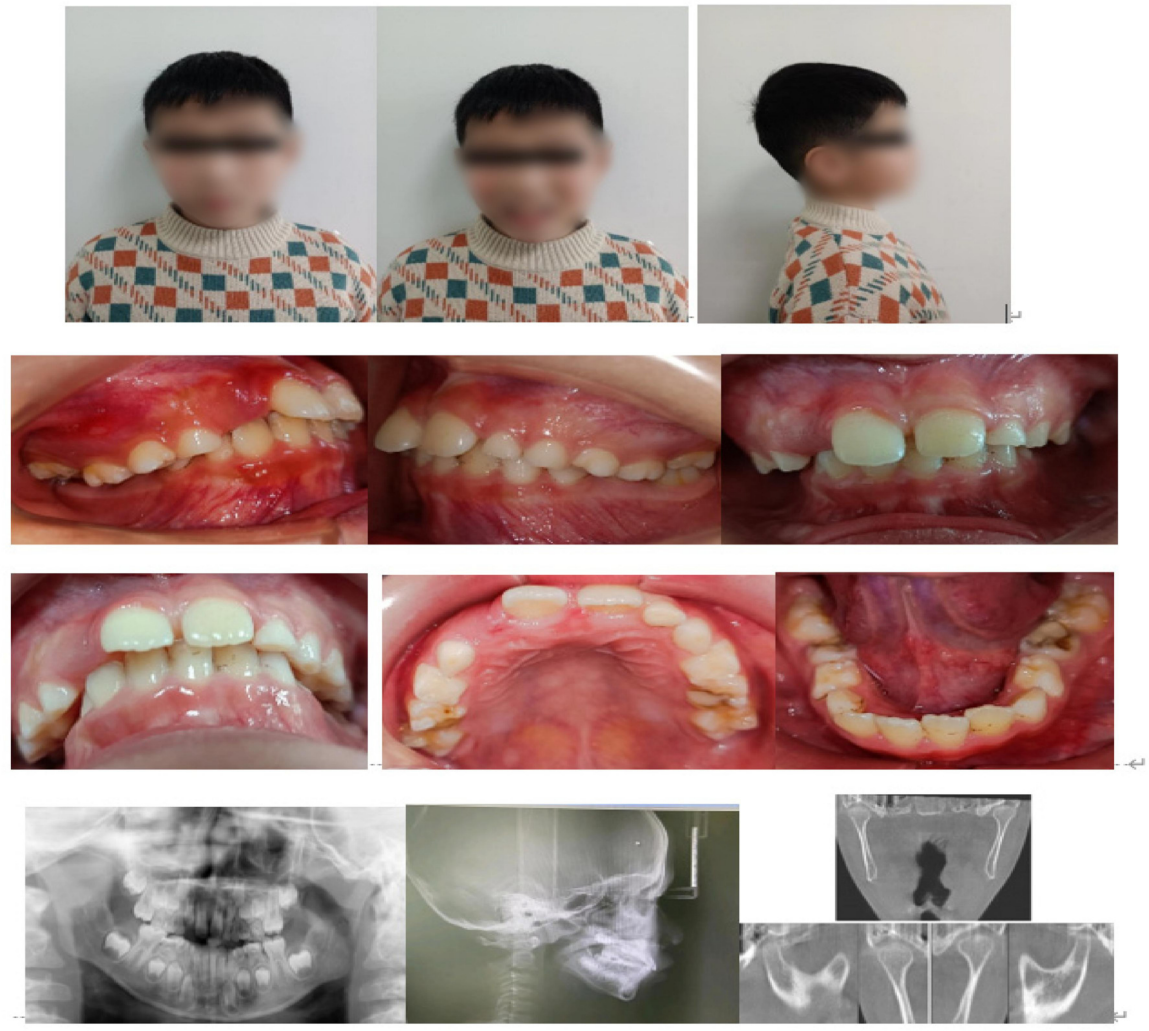
Extraoral frontal view, intraoral occlusal view and radiographic images before treatment.

Pre-treatment cephalometric measurements (2023): SNA = 81.7°, SNB = 77.64°, ANB = 4.06°, U1-SN = 101.93°, L1-MP = 93.93°, FH-MP = 29.05°, presenting neutral vertical facial type. The lingual inclination angle of the right mandibular first molar was 24.6° before treatment (Figure 2).

### 2.3 Diagnosis

Skeletal Class I malocclusion;
Dental unilateral right posterior severe scissors bite;
Constricted mandibular dental arch, mandibular midline deviation;
Mixed dentition with partial primary molar secondary caries;
Tendency of high-angle facial type and mandibular clockwise rotation.

### 2.4 Treatment objectives

Primary objectives: Correct right posterior scissors bite; align and level upper and lower dental arches; improve anterior overjet and overbite; correct mandibular midline deviation; treat secondary caries of primary molars.

Secondary objectives: Expand constricted mandibular arch; upright lingually inclined molars; maintain vertical facial height; inhibit mandibular clockwise rotation; preserve long-term TMJ stability.

### 2.5 Treatment plan

1. Adopt 0.75 mm thick clear aligners to level and align dental arches, correct mandibular midline deviation;
2. Design alternating intermaxillary traction for severely inclined 85 and 46 to release posterior scissors bite and remodel mandibular arch morphology;
3. Resin buccal attachments and lingual traction clasps were bonded for traction anchorage; traction rubber band: 2 ounces, 5/16 inch, wearing no less than 20 h per day;
4. Replace a new set of aligners every 10 days; arrange restorative treatment for secondary caries of 65, 75 and 85 after orthodontic treatment;
5. Retention protocol: Full-time wear of transparent retainer for 6 months post-treatment, followed by nocturnal wear for another 12 months.

A complete digital workflow was utilized for fabricating the clear aligners. High-precision intraoral scanning was performed to obtain three-dimensional dentition data, replacing conventional impression taking. Standard STL digital models were generated, and aligners were thermoformed from medical-grade thermoplastic sheets via computer-aided manufacturing. This digital approach improved fabrication accuracy, adaptation, and predictability of tooth movement.

### 2.6 Treatment process

Treatment timeline: Initial consultation (March 2023) → Aligner delivery (April 2023) → First traction adjustment (June 2023) → Treatment interruption due to relapse (August 2023) → Treatment resumption (September 2023) → Treatment completion (December 2023).

From phase 1 to 16 of aligner wearing, mandibular molars were distalized cooperated with arch expansion, combined with alternating traction of 16 and 46 to release scissors bite and adjust dental midline. At phase 8, the scissors bite between 16 and 46 was relieved, but occlusal coverage was insufficient; the scissors bite of 54 and 84 remained uncorrected. Lingual clasps were additionally bonded on 54 and 84 for auxiliary alternating traction. Until phase 16, the right posterior scissors bite was mostly relieved, but stable occlusal contact of 54 and 84 was not established.

Subsequently, treatment was interrupted for 62 days during summer vacation with poor compliance: no retainer worn, daily aligner wearing time less than 4 h. Re-examination after 2 months showed that the occlusion of 16 and 46 returned to normal, while 54 and 84 relapsed to complete scissors bite, and occlusal coverage of 55 and 85 became shallow.

After treatment resumption, revised alternating traction was designed for 54–84 and 16–46. Traction was not arranged for 55 and 85 due to partial crown defect. Standard 2 ounces 5/16 inch rubber bands were used for intermaxillary alternating traction to further correct scissors bite and refine midline alignment. After 3 months of continuous treatment, the right posterior scissors bite was completely corrected, anterior overjet and overbite improved, and upper and lower anterior midline aligned symmetrically.

Throughout the treatment, patients were instructed to perform standardized tooth tapping training: gentle occlusal contact for 30 s followed by muscle relaxation, 30 times per set, 3 sets daily. The training was applied to counteract posterior molar elongation induced by traction, control mandibular plane angle and inhibit clockwise rotation.

### 2.7 Treatment outcomes

After treatment, upper and lower dental arches were well aligned; 85 and 46 were completely upright; right posterior scissors bite was fully corrected; constricted mandibular arch was expanded significantly; anterior overjet and overbite recovered to normal range; upper and lower dental midline were coincident.

Post-treatment cephalometric data: U1-SN = 104.98°, L1-MP = 95.12°; the lingual inclination angle of right mandibular first molar was corrected to 5.2°; mandibular arch width increased by 3.8 mm; midline deviation was completely corrected. The patient's masticatory efficiency improved significantly without occlusal discomfort or TMJ pain. Stable occlusal relationship was established without occlusal interference. No adverse complications such as root resorption, periodontal damage or TMJ dysfunction occurred during treatment. The post-treatment outcomes are presented in Figures 4–6.

**Figure 3:**
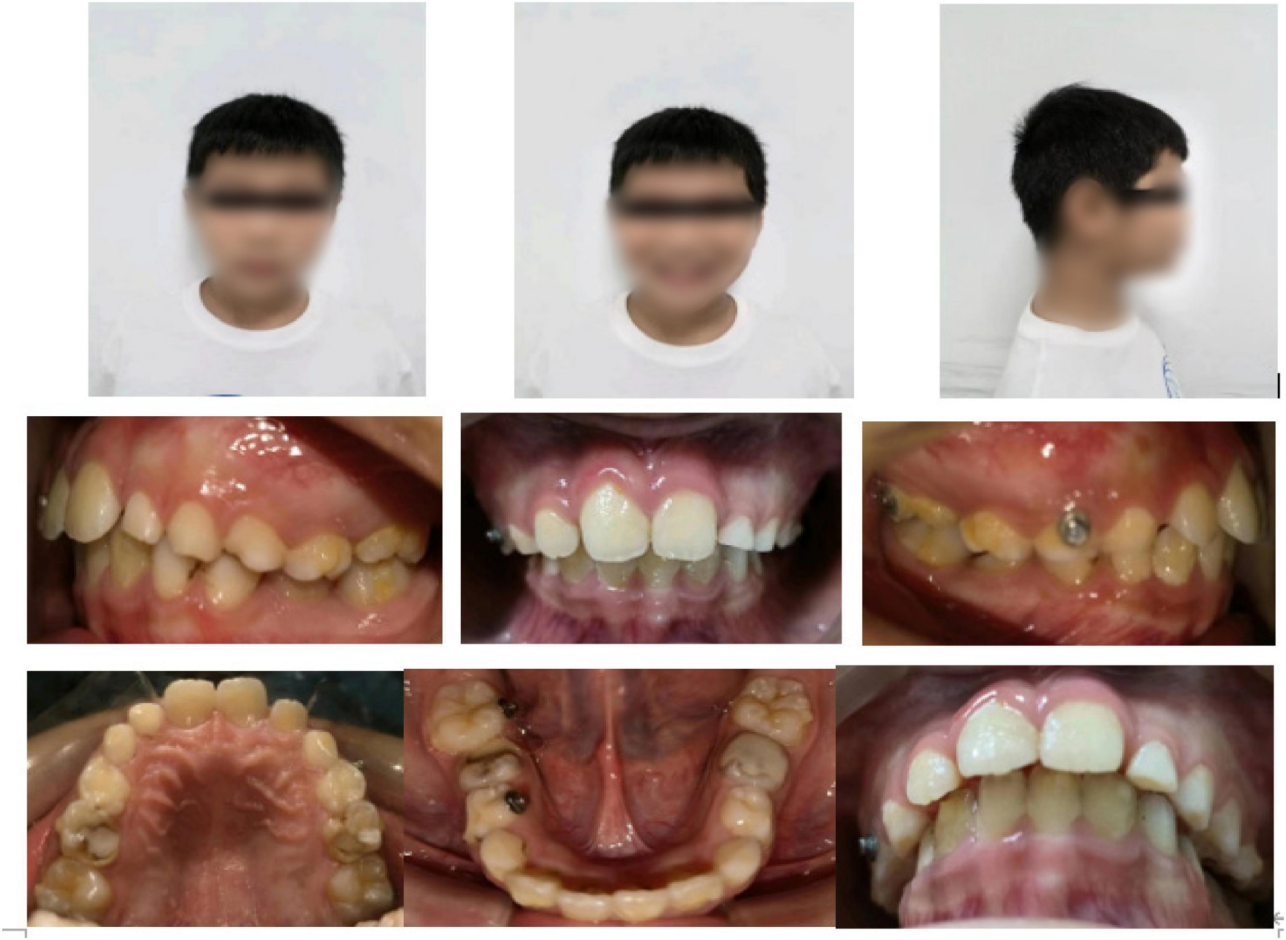
Extraoral and intraoral images during orthodontic treatment.

Figure 3: Extraoral frontal view, intraoral occlusal view and radiographic images after treatment
Cephalometric superimposition and angular changes:
Maxilla: SNA decreased slightly from 81.7° to 81.47°. Cephalometric superimposition showed maxillary position remained stable without obvious protrusion or retrusion, indicating normal maxillary growth.
Mandible: SNB decreased from 77.64° to 77.12°, with overall posterior and inferior displacement of the mandible. FH-MP increased from 29.05° to 30.76°, and the anterior-posterior facial height ratio decreased from 59.25 to 57.02, suggesting obvious mandibular clockwise rotation and progressive high-angle facial tendency.
Dentition: Upper central incisors presented mild labial inclination (U1-SN: 101.9°→104.98°); lower central incisor inclination remained stable (L1-MP: 93.93°→95.12°) with favorable dental compensation.

**Figure 4:**
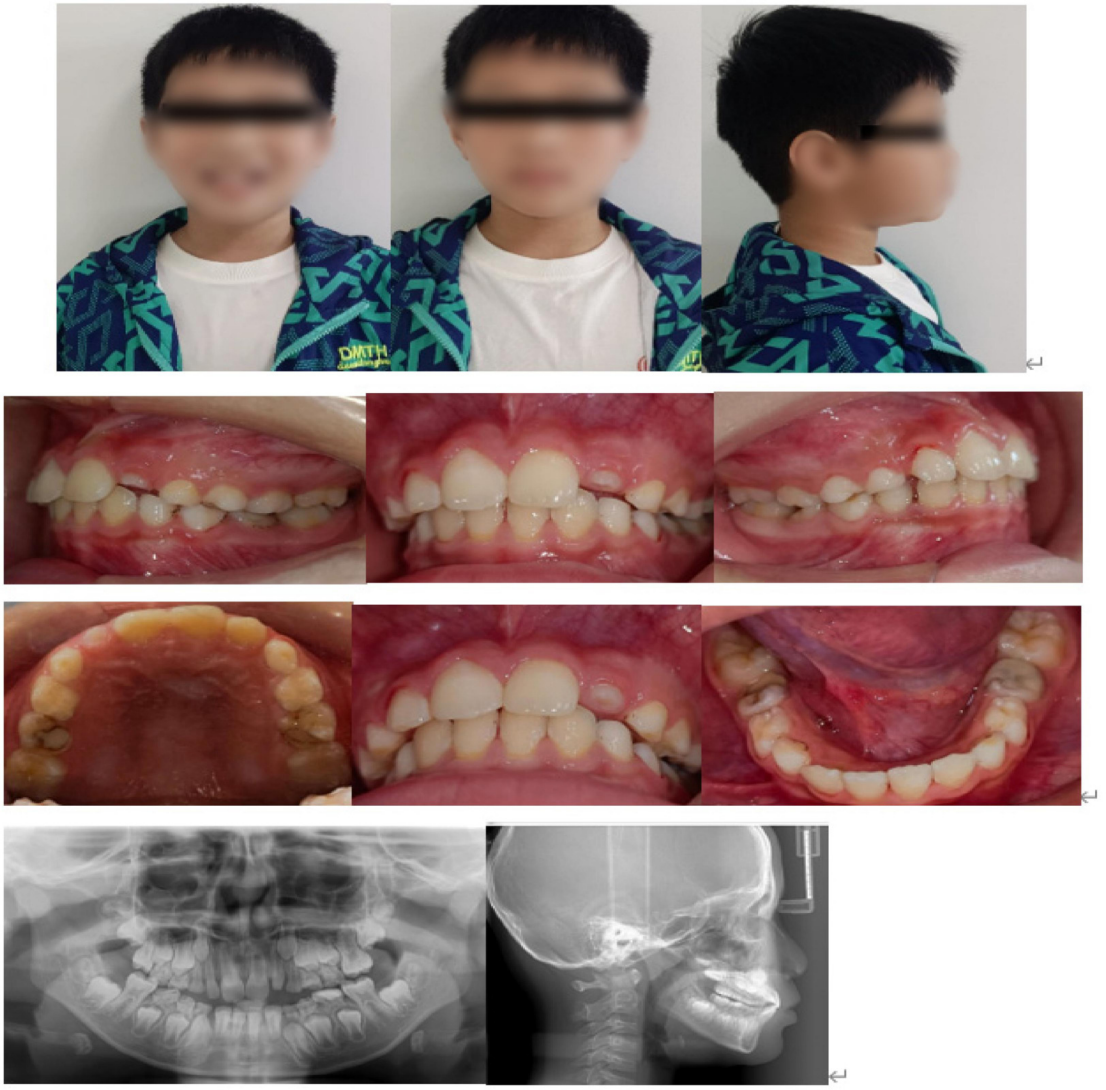
Post-treatment facial view, intraoral view, and x-ray film.

**Figure 5:**
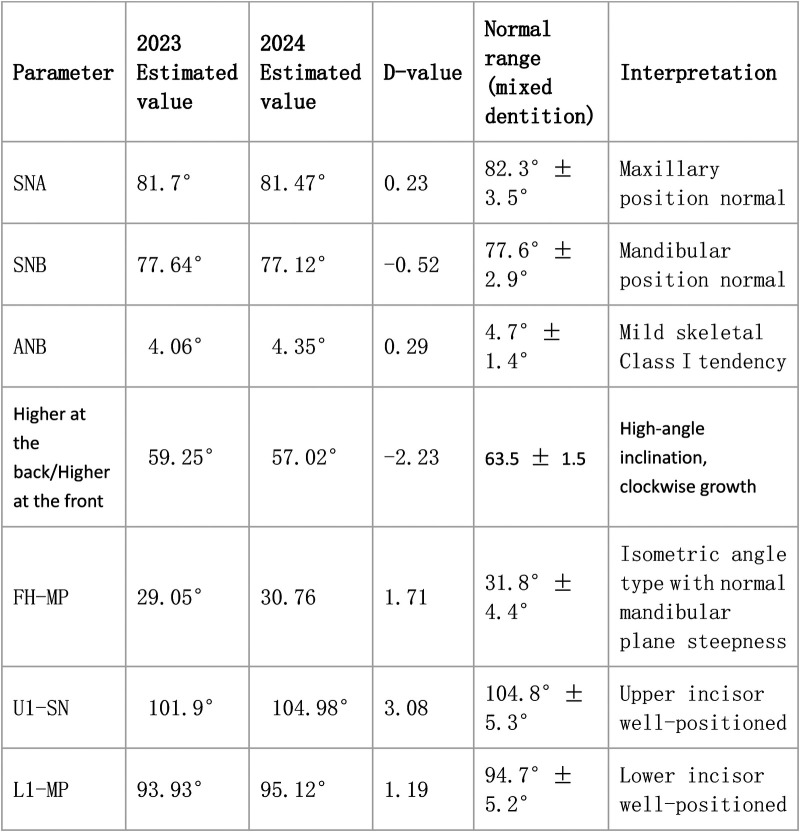
Comparison of craniofacial cephalometric parameters between 2023 and 2024.

**Figure 6:**
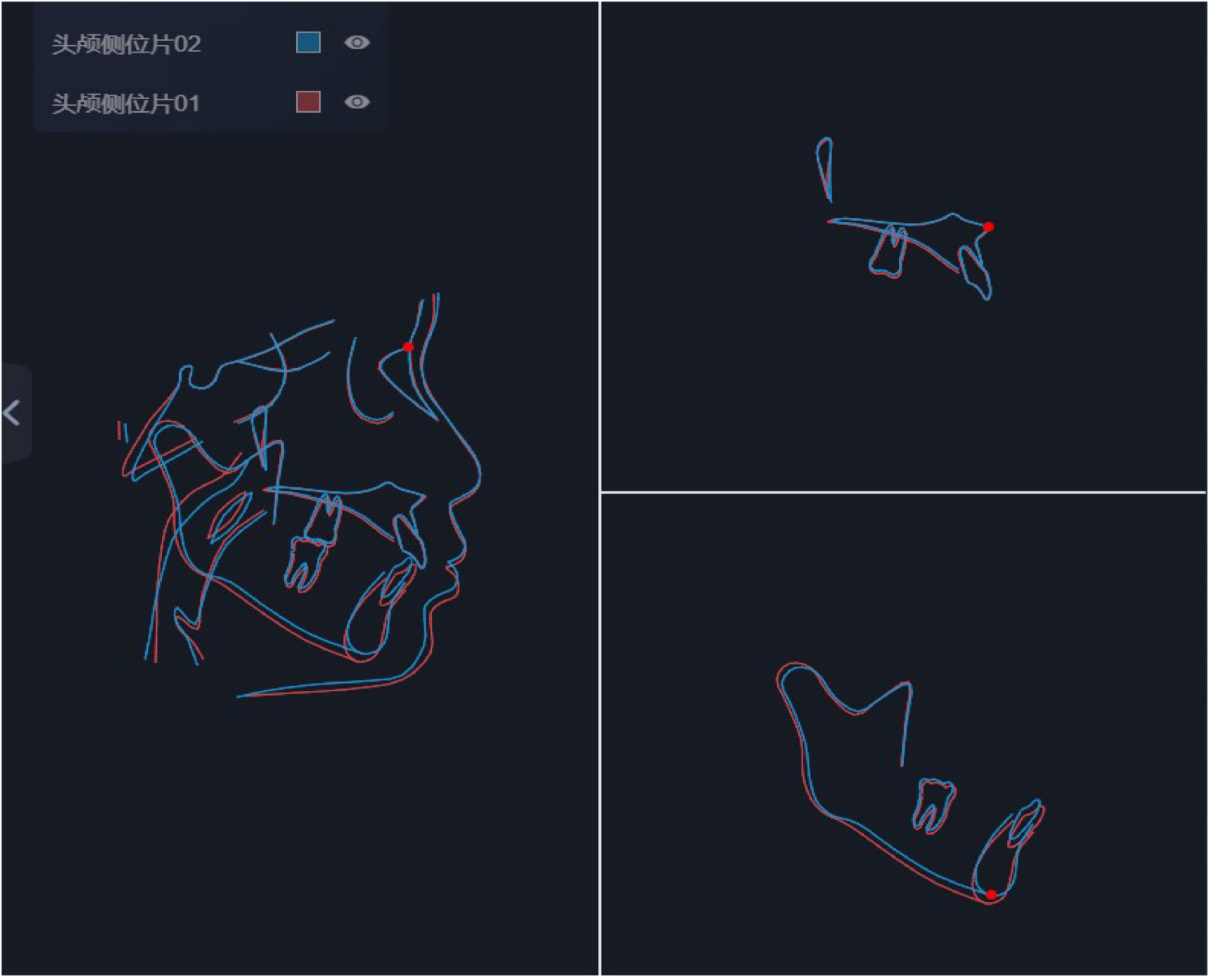
Cephalometric superimposition (red line: 2024; blue line: 2023).

### 2.8 Retention protocol and follow-up scheme

#### 2.8.1 Stage 1: Intensive retention (0–12 months after treatment)

Wear modified removable retainer full-time in daytime (removed only during meals and toothbrushing); wear transparent vacuum-formed retainer at night.

Objectives: Control posterior vertical height, inhibit progressive mandibular clockwise rotation and high-angle deterioration; stabilize axial inclination of anterior teeth, prevent excessive labial inclination of upper incisors and compensatory lingual inclination of lower incisors; correct oral bad habits such as mouth breathing and abnormal swallowing.

#### 2.8.2 Stage 2: Gradual reduction retention (12–24 months after treatment)

Reduce daytime retainer wearing; only wear removable retainer 5–7 nights per week, with transparent retainer for daytime maintenance. Re-examination every 3 months, adjust wearing frequency according to occlusal stability and cephalometric changes.

Objective: Reduce retainer dependence gradually and continuously monitor vertical skeletal growth trend.

#### 2.8.3 Stage 3: Long-term maintenance (24 months to permanent dentition eruption)

Only nocturnal wear of transparent retainer, at least 3–5 nights per week until all permanent teeth erupt and occlusion stabilizes. After complete permanent dentition formation, select fixed lingual retainer or continued transparent retainer according to individual condition.

Objective: Prevent dental drift and interdental space relapse during mixed dentition transition; long-term monitor jaw growth direction and restart early intervention if necessary.

## 3 Cephalometric superimposition analysis

Maxillary structure: The maxillary contour of 2023 (blue line) and 2024 (red line) was almost overlapped with negligible difference, consistent with the slight change of SNA angle. The maxilla maintained stable anteroposterior position without abnormal growth deviation.
Mandibular structure: The most obvious changing region. The 2024 mandible displaced obviously posteriorly and inferiorly with a flatter inferior border; the mandibular plane became steeper, which was consistent with the increased FH-MP angle. It confirmed mandibular clockwise rotation with increased lower facial height and typical high-angle growth tendency.
Anterior teeth: Upper central incisors showed slight labial inclination in 2024, corresponding to the increased U1-SN angle; lower incisor position remained stable without obvious axial change. Molar position had no significant mesial or distal drift, with overall occlusal stability.
Soft tissue profile: The chin contour of 2024 was posterior and inferior, with increased lower facial height, presenting elongated facial morphology and aggravated chin retrusion visual performance. Uncontrolled high-angle tendency combined with mandibular clockwise rotation may further induce open bite, deep overbite and upper airway stenosis, requiring long-term growth monitoring and interceptive control.

## 4 Discussion

For children with unilateral posterior scissors bite, pre-treatment evaluation should prioritize TMJ functional assessment (2). It is necessary to detect mouth opening range, opening path, joint clicking and tenderness; if abnormality exists, CBCT or MRI should be performed to rule out organic structural lesions. In this case, no TMJ symptoms were found clinically, and pre-treatment CBCT confirmed symmetrical condylar morphology without organic lesions.

Although recent meta-analyses have reported that the relapse rate of severe scissors bite with clear aligners may be higher than with fixed appliances, clear aligners were still selected for this mixed dentition patient for the following reasons: (1) The mixed dentition stage involves exfoliating primary teeth and erupting permanent teeth; fixed appliances are difficult to adapt to continuous dentition changes and may cause mucosal irritation, enamel demineralization, and poor oral hygiene in children. (2) Clear aligners are removable, comfortable, aesthetic, and more cooperative for children. (3) This case mainly exhibited dentoalveolar scissors bite without severe skeletal discrepancy, which was suitable for sequential tooth movement via clear aligners. (4) The risk of relapse could be reduced by strengthened compliance control, extended retention, and long-term growth monitoring. Therefore, clear aligners were more appropriate for early interceptive treatment in this child.

Unilateral posterior scissors bite can be corrected by intermaxillary alternating traction, MEAW archwire technique and micro-implant anchorage (3). Most clinical studies focus on single-tooth crossbite correction, while severe unilateral multi-tooth posterior scissors bite is relatively rare with high orthodontic difficulty. Compared with traditional fixed appliances and MEAW technique, clear aligners have superior comfort and avoid mucosal irritation, which is more suitable for young children (4).

Recent meta-analyses have demonstrated that clear aligner treatment for severe scissors bite presents a higher relapse rate compared with fixed orthodontic appliances, which is consistent with general orthodontic clinical findings. However, considering the patient's mixed dentition stage and the unique physiological characteristics of growing maxillofacial structures and developing dentition, clear aligner therapy was preferentially selected for this case based on comprehensive assessments of clinical safety, oral adaptability, and treatment feasibility. The detailed rationales are as follows. First, the mixed dentition stage is characterized by the coexistence of primary and permanent teeth with dynamically developing and replacing dentition, accompanied by delicate and highly plastic alveolar bone and periodontal tissues. Traditional fixed appliances consist of rigid components including brackets, bands, and archwires, which may cause persistent friction and irritation to immature oral mucosa, frequently resulting in mucosal erythema and recurrent oral ulcers. Moreover, fixed appliances tend to accumulate dental plaque and food debris. Given children's limited oral hygiene compliance during the mixed dentition period, such appliances significantly increase the risk of enamel demineralization, dental caries, and gingivitis in both primary and newly erupted permanent teeth. In contrast, clear aligners feature smooth and flexible surfaces and are removable, effectively avoiding mucosal stimulation, improving wearing comfort, and enhancing children's treatment cooperation. Second, the core objectives of orthodontic intervention in mixed dentition are to guide normal maxillofacial growth, correct functional occlusal disorders, and create sufficient space for permanent tooth eruption, rather than achieving aggressive tooth movement with heavy anchorage. The severe scissors bite in this case was primarily attributed to functional and dento-occlusal abnormalities without severe skeletal deformity. The lightweight and progressive tooth movement mode of clear aligners can gently correct occlusal malformations, adapt to the dynamic growth characteristics of mixed dentition, and prevent potential adverse effects of excessive force from fixed appliances on immature alveolar bone and permanent tooth germs. Third, continuous tooth replacement and dynamic dentition changes during the mixed dentition require frequent adjustment and re-bonding of fixed appliances, which is operationally cumbersome and may damage tooth enamel. Digital clear aligner therapy allows staged updates of treatment protocols and aligners according to the progression of primary tooth exfoliation and permanent tooth eruption, achieving personalized and flexible treatment adaptive to developmental dentition changes.

In this case, the right posterior multi-tooth scissors bite was complicated with severe lingual inclination of 85 and 46, as well as extensive primary molar caries with hard tissue defect, further increasing treatment difficulty. Without micro-implant anchorage, clear aligners combined with simple alternating intermaxillary traction achieved ideal correction outcomes. The inherent thickness of clear aligners acted as an occlusal pad to lift posterior teeth vertically, create space for molar uprighting and release scissors bite lock (5, 6).

The core difficulty of this case is the uprighting of severely lingually inclined molars, which easily causes posterior molar elongation and secondary mandibular clockwise rotation—unfavorable for high-angle skeletal patients. Therefore, standardized tooth tapping training was matched to counteract molar elongation, control vertical facial height and maintain skeletal vertical balance, which is worthy of clinical promotion (7).

Children in early growth period have high periodontal tissue and jawbone plasticity, with sensitive response to orthodontic force and rapid tissue remodeling. Clear aligners possess the advantages of superior esthetics, high comfort and easy oral cleaning, which are widely applied in early interceptive orthodontics (8, 9). This case verified that clear aligners combined with alternating traction can effectively correct severe unilateral posterior scissors bite in mixed dentition, with simple operation and reliable curative effect.

### 4.1 Treatment limitations

Short follow-up period: Only immediate treatment outcomes were observed, lacking 6-month and 1-year long-term follow-up to evaluate occlusal long-term stability;

Patient compliance limitation: Poor wearing compliance during treatment interruption directly led to scissors bite relapse, which is an inevitable limiting factor for pediatric clear aligner treatment (10).

Restricted tooth condition: Extensive caries and hard tissue defect of tooth 85 reduced tooth anchorage capacity, limiting the magnitude of orthodontic force and prolonging tooth movement cycle;

Absence of micro-implant anchorage: No skeletal anchorage was applied for severely inclined molars, resulting in prolonged correction duration.

### 4.2 Literature comparative analysis

Wei et al. (2) confirmed that unilateral posterior crossbite is often accompanied by condylar morphological asymmetry; this case presented purely dental scissors bite with symmetrical condyle, indicating better prognosis than skeletal displacement cases. Duan et al. (3) reported that traditional fixed intermaxillary traction is mostly applied in permanent dentition crossbite, while this study adopted clear aligners suitable for mixed dentition children with better esthetic performance.

Recent meta-analysis confirmed that clear aligners have favorable efficacy in dental malocclusion correction, but the relapse rate of severe scissors bite is higher than fixed appliances, which is consistent with the relapse characteristic of this case (11). Multiple 2023–2025 studies have shown that clear aligners are reliable for early interceptive orthodontics in mixed dentition, especially for arch expansion and posterior crossbite correction, but compliance management and long-term retention must be emphasized (6, 7, 9).

### 4.3 Patient subjective evaluation

The child and guardians reported no foreign body sensation of clear aligners, convenient for diet and oral cleaning. Traction rubber bands were easy to wear with mild discomfort. After relapse, parental supervision was strengthened and subsequent treatment compliance improved significantly. The child had no psychological inferiority due to orthodontic appliances and was highly satisfied with esthetic and functional outcomes.

## Data Availability

The original contributions presented in the study are included in the article/Supplementary Material, further inquiries can be directed to the corresponding author.
